# Application of CRISPR genetic screens to investigate neurological diseases

**DOI:** 10.1186/s13024-019-0343-3

**Published:** 2019-11-14

**Authors:** Raphaella W. L. So, Sai Wai Chung, Heather H. C. Lau, Jeremy J. Watts, Erin Gaudette, Zaid A. M. Al-Azzawi, Jossana Bishay, Lilian Tsai-Wei Lin, Julia Joung, Xinzhu Wang, Gerold Schmitt-Ulms

**Affiliations:** 1 Department of Laboratory Medicine & Pathobiology, University of Toronto, Medical Sciences Building, 6th Floor, 1 King’s College Circle, Toronto, Ontario M5S 1A8 Canada; 2 Tanz Centre for Research in Neurodegenerative Diseases, University of Toronto, Krembil Discovery Centre, 6th Floor60 Leonard Avenue, Toronto, Ontario M5T 2S8 Canada; 3 Department of Pharmacology & Toxicology, University of Toronto, Medical Sciences Building, 6th Floor, 1 King’s College Circle, Toronto, Ontario M5S 1A8 Canada; 4 Departments of Biological Engineering and Brain and Cognitive Science, and McGovern Institute for Brain Research at MIT, Cambridge, MA 02139 USA; 5 Broad Institute of MIT and Harvard, Cambridge, MA 02142 USA

**Keywords:** CRISPR-Cas9, Functional genetics, sgRNA, CRISIPR KO, CRISPRi, CRISPRa, Neurological diseases, Neurodegenerative diseases, Survival screens, Marker selection screens

## Abstract

The adoption of CRISPR-Cas9 technology for functional genetic screens has been a transformative advance. Due to its modular nature, this technology can be customized to address a myriad of questions. To date, pooled, genome-scale studies have uncovered genes responsible for survival, proliferation, drug resistance, viral susceptibility, and many other functions. The technology has even been applied to the functional interrogation of the non-coding genome. However, applications of this technology to neurological diseases remain scarce. This shortfall motivated the assembly of a review that will hopefully help researchers moving in this direction find their footing. The emphasis here will be on design considerations and concepts underlying this methodology. We will highlight groundbreaking studies in the CRISPR-Cas9 functional genetics field and discuss strengths and limitations of this technology for neurological disease applications. Finally, we will provide practical guidance on navigating the many choices that need to be made when implementing a CRISPR-Cas9 functional genetic screen for the study of neurological diseases.

## Background

Functional genetic screens provide a powerful discovery tool for identifying genes or genomic elements that are pertinent to a phenotype-of-interest. A few years ago, the clustered regularly interspaced short palindromic repeats (CRISPR)-associated Cas9 endonuclease system was adopted for this purpose to reveal a wealth of mechanistic insights, from drug resistance in cancer to neuronal toxicity in amyotrophic lateral sclerosis.

Prior to CRISPR-Cas9, functional genetic screens employed RNA interference (RNAi) oligonucleotides for loss-of-function studies and cDNA overexpression libraries for gain-of-function studies [1, 2]. However, RNAi-based screens reduce gene expression at the transcript level, making residual expression a perpetual concern, and cDNA overexpression libraries are challenging to construct. Side-by-side comparisons with RNAi knockdown analyses revealed additional compelling advantages to using CRISPR-Cas9 for functional genomic knockout screens, including fewer false positives and considerable gains in signal-to-noise ratios [3].

The CRISPR-Cas9 system was initially discovered as an adaptive immune system in prokaryotes against phages [4, 5]. Although many CRISPR systems have been described in recent time, this review will focus on the type II CRISPR system engineered from *S. pyogenes,* as it is the most widely-used platform for conducting functional genetic screens. Cleavage by *S. pyogenes* Cas9 requires an NGG protospacer adjacent motif (PAM) recognition site immediately following the 3′ end of a 20 nucleotide protospacer sequence to generate a double-stranded break (DSB) three bases upstream of the 3′ end of the protospacer.

DSBs are repaired by endogenous host cell mechanisms, namely non-homologous end joining (NHEJ) or homology-directed repair (HDR). NHEJ is error-prone and leads to insertions or deletions (indels) near the cut site. Consequently, indels can cause frameshift mutations, which may alter peptide sequences or result in premature stop codons [6]. In most instances, transcribed mRNAs with premature stop codons are degraded through non-sense mediated decay, effectively resulting in a gene knockout (KO). In contrast, HDR is a high-fidelity repair program that can be used to integrate desired genomic modifications. Various methods have been shown to enhance the efficiency or shift the relative engagement of host-encoded HDR versus NHEJ programs [7]. These include synchronizing the cell cycle, altering the expression of key proteins that modulate homologous recombination, or offering single-stranded or double-stranded donor DNA for directing the enzyme to the DSB repair site. Similarly, Cas9 mutants have been developed that increased specificity [8–10]. In one implementation, a Cas9 mutant was derived that not only improved specificity but also broadened the PAM sequence compatibility [11]. Two very recent studies expanded the repertoire of genome-editing tools by CRISPR-associated transposases from *Vibrio cholerae* (TN6677) [12] and *Scytonema hofmanni* (ShCAST) [13] with favorable characteristics for precise gene editing applications. Both systems allow RNA-guided DNA insertions at high frequencies and bypass the need for homology-directed repair.

Whereas early uses of CRISPR-Cas9 technology were mostly for single-gene applications, CRISPR has since been adapted to target multiple genes simultaneously (multiplexing) by pooling sgRNAs [14, 15]. Unlike other genome editing tools, e.g., zinc finger nucleases (ZFNs) and transcription activator-like effector nucleases (TALENs), which require time-consuming customization of DNA binding proteins, the use of sgRNAs is more technologically feasible and cost-efficient. Packaging sgRNAs on a large scale for genetic screens is also considerably easier than packaging DNA binding proteins. Thus, by reducing both costs and logistical barriers, CRISPR-Cas9 has become an attractive modality for functional genetics research [16, 17]. Different groups have combined orthologs of Cas9 or Cpf1, another RNA-guided endonuclease of the CRISPR-Cas9 system, to achieve multiplexed screens. Unlike Cas9, which requires RNase III and additional Cas proteins to process polycistronic guide precursors, Cpf1 is self-sufficient in its ability to process CRISPR arrays. Hence, instead of having only one sgRNA per vector, one can package multiple sgRNAs targeting the same gene in a single vector for Cpf1, effectively reducing the technical burden [18–20].

**Fig. 1:**
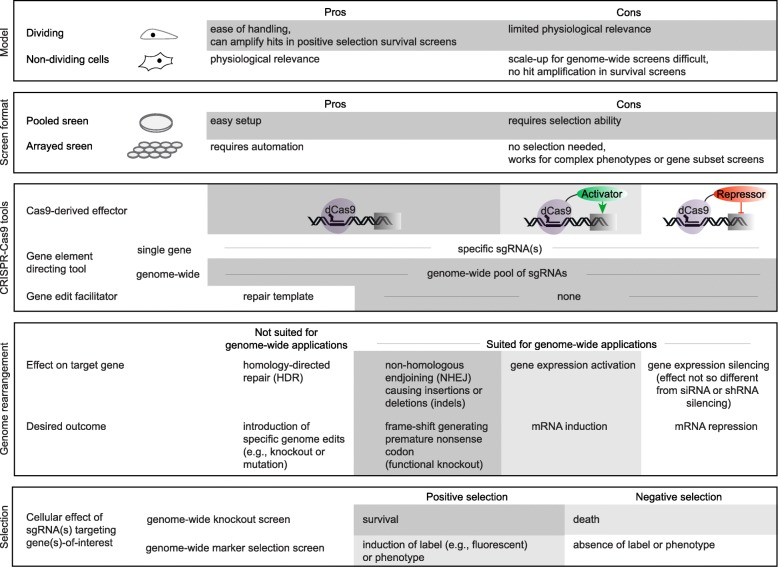
Overview of CRISPR-Cas9 functional genetics applications. Due to the inherently modular nature of CRISPR-Cas9 genome editing, there are many ways to implement a functional genetics screen based on this technology. Common choices realized in published work are highlighted in this figure in darker grey shading

In addition to CRISPR-Cas9 knockout (CRISPR KO) screens, CRISPR-Cas9 technology has also been adapted to genome-scale transcriptional inhibition or activation screens (Fig. 1). Transcriptional modulation uses deactivated Cas9 (dCas9), which has mutations in both the RuvC and the HNH nuclease domains. When paired with sgRNAs directing it to the promoter or regulatory sequences of a gene, dCas9 does not cleave DNA. To induce transcriptional inhibition (CRISPRi) or activation (CRISPRa), dCas9 is fused to repressor (e.g., KRAB) or activator (e.g., VP64) domains, respectively [21, 22]. Whereas early CRISPRa complexes had only one activator domain, current derivatives, like the synergistic activation mediator (SAM), rely on the fusion of multiple activator domains (e.g., VP64, MS2 bacteriophage coat proteins, NF-kB trans-activating subunit p65, or an activation domain from human heat-shock factor 1) to achieve more robust gene activation [22, 23]. Unlike cDNA libraries that rely on heterologous transgene expression, CRISPRa modulates gene expression at the endogenous gene transcription level [1, 23]. In principle, CRISPRi screens are similar to CRISPR KO screens because both reduce or eliminate gene expression. However, whereas CRISPR KO causes permanent gene expression ablation, CRISPRi mediates a reversible expression deficiency [24]. Generally, CRISPRi mimics RNAi based approaches better than CRISPR KO applications. Also, when working with cancer cell models that often feature increases in genomic copy number or chromosomal rearrangements characterized by the presence of amplified regions, sgRNA-directed CRISPRi offers an attractive alternative to CRISPR KO. In these karyotype-perturbed cells, CRISPR KO can cause an excessive number of DSBs, which may kill the cells, thereby leading to false positives in essential gene analyses [25–27].

The following sections will discuss the design considerations and methodology of CRISPR-Cas9 functional genomics screens, from selecting a suitable model and conducting the screen, to analyzing data and validating candidates. We will put a spotlight on reports that paved the way for some of the most exciting new applications. Finally, we will emphasize early implementations in the neurological disease research area and discuss their strengths and limitations. Throughout, we will provide guidance on how to navigate around limitations and pitfalls when planning a CRISPR-Cas9 functional genetic screen for the study of neurodegenerative diseases.

In order to manage the length of this report, we had to make tough choices in our handling of a body of literature that is not only growing fast but is also characterized by a high volume of excellent reports. A report of this length cannot do justice to the outstanding work of many colleagues, and we apologize if we failed to reference pertinent works. In addition to citing the primary literature, our selection of references was guided by the wish to emphasize reports that provide useful background or detailed technical advice and as such complement a review that focuses on concepts and design choices of CRISPR-Cas9 functional genetic screens.

## Main text

### Choosing a model system

The most appropriate design of a CRISPR-Cas9 functional genetic screen depends on the research question, the existence of a robust phenotype-of-interest, and a paradigm in which it can be studied. In vitro cell models are selected when scalability trumps the need for physiological authenticity, ex vivo models offer a compromise in this regard, and in vivo models are indispensable when such a compromise cannot be made.

#### Cell lines

To date, most genome-scale CRISPR-Cas9 functional genetic screens have been undertaken with dividing immortalized cell lines that can be easily scaled. A critical advantage of these models represents the ease with which they can be engineered to express a phenotype-of-interest. For example, a reporter, such as the enhanced green fluorescent protein (EGFP), can be fused to a gene product of interest [28]. The proliferative nature of immortalized cells also facilitates clone formation in positive selection survival screens. The availability of these clones, which can be saved as stocks, not only constitutes a useful resource but also alleviates concerns that information is irretrievably lost during downstream processing steps [29]. Since cell clones can provide unlimited genomic DNA, their use can increase the reliability of DNA sequencing data, whether it is sequencing the genome-embedded sgRNA or the target gene to assess genetic editing. It is worth noting that the choice of proliferating cell line matters. Cancer cell lines, which are aneuploid, are susceptible to additional non-target toxicities from CRISPR-Cas9 KO editing (see below) [25]. Other factors to consider when working with immortalized cell lines are that results don’t translate to a more physiological system, chiefly because the need for non-stop division may preclude certain phenotypes (e.g., the accumulation of protein aggregates) and the clonal variability that one might observe. Their abnormal gene expression profile can also limit the physiological relevance of experimental findings. The need to replicate findings in a more physiologically relevant model has led investigators to make use of dividing cells for their initial screens but move to neurons or other primary cells for secondary validation [30, 31].

#### ESC- and iPSC-derived neural cells

A workaround to some of the limitations of immortalized cell lines is to work with embryonic stem cells (ESCs) or induced pluripotent stem cell (iPSC)-derived neural cells. ESCs and iPSCs can be expanded in culture to achieve high cell numbers before being differentiated into neurons. This characteristic makes them more authentic than cell lines and more amenable to higher throughput library screens than primary cells (see below). Due to their diploid genome, ESCs and iPSC are less prone to genomic drift than aneuploid immortalized cell lines, which tend to diversify during extended cell culture. This feature of ESCs and iPSCs facilitates the engineering of isogenic cells that differ only in a specific gene-of-interest. A popular implementation of this experimental design is to compare side-by-side wild-type and mutated cells that carry sequence variants associated with familial neurodegenerative diseases [32]. A limitation of ESC- or iPSC-derived neurons is that these neurons tend to remain immature and resemble fetal neurons. Although partially ameliorated through co-culture with astroglia, these neurons exhibit, for instance, little spontaneous electrical network activity [33]. Also, relative to working with immortalized cell lines, the generation of ESC- or iPSC-derived neural cells requires considerable resources and investments in time due to the need to generate, sort, and differentiate the cells.

#### Primary cells in culture

Since immortalized cells have often undergone profound genomic rearrangements, and ESC- or iPSC-derived neurons may not exhibit authentic features, observations need to be interpreted with caution unless verified in models with greater physiological relevance. In this regard, primary neural cell cultures can be more useful [34, 35]. However, primary neurons often undergo cellular senescence and death under ex vivo culture conditions [36], a phenomenon ascribed to the lack of authentic molecular and cellular stimulation that persists in two-dimensional cultures.

If the experimental endpoint entails increased expression of a reporter gene or accumulation of toxic protein aggregates, then the short lifespan of neuronal cultures may not pose a meaningful concern. However, if the objective is to study the gradual process of mammalian neurodegeneration, the experiment must be carefully designed to ensure the life-or-death phenotype occurs within this viability window. Thus, higher concentrations of toxins are often used. For instance, concentrations of Aβ aggregates used for studying Alzheimer’s disease in culture are commonly higher than physiological levels, which may reduce the translational relevance or applicability of the results [37–39]. Neurons are often cultured in medium with supplements, e.g., superoxide dismutase and glutathione, to extend their lifetime. Although such media supplements can protect cells against oxidative stress, they may also render the models resistant to the study of cellular degeneration.

Because of the hurdles to scalability, primary neurons in culture are less attractive for primary genome-scale CRISPR-Cas9 functional genetics screens but may come to use in more focused validation screens. Glia cells, whose contributions to the pathobiology of these diseases are increasingly appreciated, may offer a more tractable target for these kinds of screens due to their proliferative nature.

A less obvious confounder of primary cells arises from the interactions between neighboring cells. For instance, neuroinflammation and cellular senescence in one cell have been shown to induce death in a neighboring cell [40]. Thus, the phenotype presentation may not necessarily be linked to the sgRNA received by each individual cell, confounding screen results. In these situations, an arrayed screen can ensure that the cell fates are directly caused by the transduction of a single sgRNA [41].

Moreover, in culture, even primary cells lose some of the authentic biology present in the brain as recently documented with cultured microglia, which exhibited profoundly different molecular signatures of expressed genes and microRNAs when compared with in vivo microglia [42]. Finally, primary neural cells derived from animals differ genetically from human cells and therefore do not necessarily recapitulate cellular disease-phenotypes observed in human neurodegenerative disease.

#### In vivo models

Many animal models are available that recapitulate inherited, drug-induced and infectious neurological disease phenotypes [34]. For functional screening in in vivo neurological disease models, the challenge is to deliver sgRNAs to brain cells, accomplished through adeno-associated viruses (AAV). If the targets are native brain cells, the need to differentiate transduced from untransduced cells requires the co-delivery of a selection marker (e.g., EGFP). Due to the relatively small packaging limit of AAVs, the host also preferably needs to already express Cas9 [43].

Implementation of an in vivo screen is easier if there is no need to target native brain cells but rather brain tumors. In the latter case, cells can be targeted ex vivo prior to their transplantation. A glioblastoma screen in Cas9 mice undertaken to target 49 genes (each with 5 sgRNAs) associated with tumor formation and resistance to temozolomide—a first-line treatment for glioblastoma multiforme—represents an example of this design [43].

In vivo models remain the gold standard for hit validation in functional genomics analyses. For instance, short-listed gene products that appeared to confer resistance to alpha-synuclein toxicity in a primary screen were validated in a rodent model of pathological alpha-synuclein transmission [44]. Nonetheless, investigators need to remain conscious of the fact that animal models do not necessarily authentically recapitulate the spatiotemporal expression of gene products-of-interest observed in human disease.

Finally, a widely applicable experimental paradigm that is applicable to more than one model system is based on the exposure to toxic neurodegenerative disease proteins (e.g., oligomeric Aβ) [34]. This approach can be easily implemented with cells in culture but is also available for in vivo work when, e.g., a rodent model has been engineered to overexpress, produce and/or secrete a toxic protein-of-interest.

### Design considerations and methodology

**Fig. 2:**
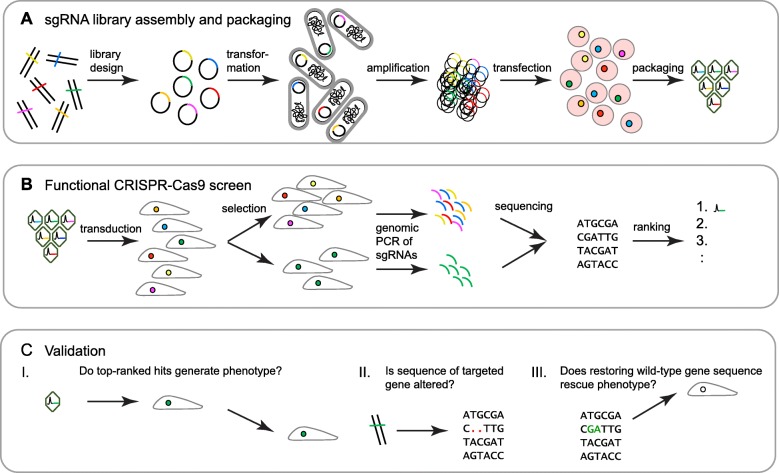
Workflow of CRISPR-Cas9 functional genetic screens. **a** sgRNA libraries are ligated onto plasmid backbones are then transformed into electrocompetent bacterial cells. The amplified sgRNA library is purified from a bacterial lysate and transfected into virus-producing cells to generate a sgRNA library. **b** The sgRNA library is transduced into target cells, which are subsequently subjected to phenotype selection. Genomic DNA is then harvested, and embedded sgRNAs are amplified by PCR and identified by NGS. Hits are determined and ranked by their relative enrichment or depletion of the respective sgRNAs in the selected versus non-selected control cells. **c** The initial validation of screen hits typically relies on: I. small-scale repeat analysis targeting genes of interest with sgRNAs that had been used in the original screen, plus additional sgRNAs directed toward the same gene; II. genomic sequencing-based verification that the targeted gene was indeed sequence-altered; and III. verification that restoring the wild-type gene sequence rescues the selection phenotype

The implementation of CRISPR-Cas9 functional genetic screens can be broken into three phases: assembly and packaging of sgRNA libraries, execution of the actual screen, and validation of shortlisted targets (Fig. 2). The following provides a more detailed discussion of considerations and the steps to implement such a screen, along with suggestions for how to address challenges and improve the efficiency of the screen.

#### Arrayed versus pooled screens

Arrayed and pooled screens are two formats commonly used for querying the sgRNA library. Arrayed screens are constructed in multi-well plates, with each well being targeted by a distinct and known sgRNA. This type of screen is particularly useful when only a subset of potential genes is to be queried. Arrayed screens allow researchers to investigate complex phenotypes that may be refractory to selection and save costs downstream because next-generation sequencing (NGS) is not required to determine the identities of sgRNAs. However, these downstream savings may be offset by higher setup costs and the need for automation if hundreds of sgRNAs are being tested [45].

In pooled screens, lentiviral sgRNAs are mixed together and concomitantly transduced into target cells at a low MOI on a large scale. To preempt sgRNA drop-out, when sgRNAs are unknowingly removed from the library, pooled CRISPR-Cas9 functional genetic screens typically aim to maintain full library coverage by budgeting for each sgRNA to be transduced into an average of 500–1000 cells [46]. Cells are then physically selected by exploiting either a survival/death phenotype or the induction of a marker that allows hits to be separated, often by fluorescence-activated cell sorting (FACS). Since each sgRNA is flanked by identical sequences (e.g., the U6 promoter at the 5′ end and a fixed sequence scaffold flanking the sgRNA at the 3′ end), the identity of sgRNAs that have integrated into the genome of selected cell colonies can be retrieved by genomic PCR, followed by deep NGS analysis of amplicons [47].

#### sgRNA library design and construction

There are two main options for library design: premade or custom. Several premade pooled libraries, e.g., GeCKO.v2 and TKO CRISPR libraries, are accessible through public repositories (e.g., Addgene) for a small fee [48, 49]. The GeCKO.v2 library targets the coding sequences of known human genes with four sgRNAs per gene. Similarly, for gene activation screens, CRISPRa and SAM libraries have been shared by Addgene [23, 50].

Custom libraries may be assembled to investigate a particular subset of genes, to generate libraries for other species, or to target non-coding or intergenic regions [51]. The type of screen will often guide the design of the sgRNA library. Whereas one may preferentially target the ATG start codon or essential exons of the coding sequence in CRISPR KO screens, one needs to direct the sgRNAs to promoters and transcriptional regulation elements in CRISPRa/i. To uncover functional elements within non-coding regions, saturated mutagenesis with CRISPR-Cas9 can be performed by tiling sgRNAs across non-coding genomic segments [51, 52].

The specificity of sgRNAs can theoretically be predicted with one of several algorithms available for this purpose [53, 54]. Typically, these in silico methods determine sequence homology and off-target predictions to rank sgRNAs and compute a specificity score. Genome sequences with similarities to the protospacer but mismatches near its 3′ end (i.e., near the PAM) are less prone to be cut, and hence are less likely to be off-target [47, 55–58]. In addition to computing off-target specificity, various programs have found determinants that predict on-target efficiency [46, 59, 60]. Such determinants include GC content, the melting temperature of the sgDNA and the position of certain nucleotides relative to the PAM [60]. In vitro cell-based data-driven empirical methods of unbiased genome-scale off-target detection have been developed and validated to complement in silico-based approaches [61].

To further minimize the confounding effects of off-targets on results, sgRNA libraries are composed of multiple sgRNAs per gene. This built-in redundancy helps ascertain true positives from false ones by ensuring that evidence pointing toward top-ranked gene elements is corroborated by several sgRNAs. Redundancy also reduces the impact of sgRNA drop-out, which can occur during scale-up in the production stage (e.g., failure to be amplified, transformed into bacteria, produced as a virus, transduced into cells, etc.), leading to false negatives [46].

After one has determined the set of sgRNA sequences for the screen, custom sgRNA libraries can be synthesized using DNA synthesis services (e.g., those provided by Twist Biosciences, GenScript, or CustomArray) [46, 62, 63], amplified by PCR and cloned into a plasmid compatible with viral production. Since commercially available premade libraries are already provided as plasmids, studies employing them can proceed directly to the next step, namely the transformation into bacteria. The latter is best done by electroporation to generate sufficient quantities of plasmid DNA for a balanced sgRNA library representation. A quality check validating the comprehensiveness and balance of the library can be accomplished by NGS analysis of amplicons obtained with PCR primers directed to sequences that flank the sgRNAs. The same PCR primers are later used to amplify sgRNAs that have integrated into the genome of selected cell colonies [51, 64].

#### Virus production

Viruses used for sgRNA delivery should be integration-competent, e.g., lentiviruses and retroviruses. Although genomic integration may not be essential in arrayed screens in which the identity of the sgRNA added to each well can be known, it facilitates determination of the identities of sgRNAs-of-interest by genomic PCR in pooled screens. Lentiviruses can transduce both dividing and non-dividing cells, unlike retroviruses that can only transduce dividing cells. Thus, lentiviruses are better suited for conducting CRISPR-Cas9 functional genomics screens in in vivo or ex vivo neurological disease models. For in vivo applications, a non-viral method to stably introduce Cas9 may also need to be considered. This is because recombinant viral delivery methods can elicit immune responses and clearance of Cas9 transduced cells [54, 65] unless the study is conducted with immunodeficient hosts [43]. Adeno-associated viruses (AAVs), although not genome-integrating and therefore non-suitable for survival screens in proliferative host cells, offer advantages for in vivo brain delivery due to their less immunogenic nature [54]. In particular, the AAV9 serotype has been shown to have favorable tropism for murine brain applications. To produce any of the viruses, the sgRNA library is transfected into suitable host cells, e.g., HEK 293FT for its superior viral production ability, and the assembled viruses are harvested from the cell culture supernatant.

#### Transduction of host cells

To avoid host cells taking up more than one sgRNA, which would confound interpretation when several genes are being targeted per cell [66, 67], the objective is not to achieve maximum transduction efficiency. Instead, one should aim for an effective multiplicity of infection (MOI) of less than 0.3 [47, 68]. Since the transduction potency depends on both the viral preparation and the cell type that is to be transduced, the titer needs to be empirically determined, e.g., by capitalizing on the presence of antibiotic resistance genes encoded in the viral vector sequence and surveying the percentage of cells that survive antibiotic selection following transduction [69].

#### Positive versus negative selection

Unlike positive selection survival screens, in which the sgRNAs embedded in a handful of surviving cells are the “hits,” negative selection relies on inferring which sgRNAs are depleted from the large population of surviving colonies. A key negative selection screen application has been the determination of essential genes, e.g., chromatin regulators [68]. In a more generic sense, negative selection screens seek to identify genes whose sgRNA-guided, CRISPR-mediated perturbed expression sensitize cells to the selection pressure, i.e., make them more susceptible. Maintaining and sequencing the sgRNAs embedded in this population can be more error-prone, due to the aforementioned reasons that can lead to inadvertent drop-out of sgRNAs leading to false positives. Although this risk is ameliorated by using multiple sgRNAs per gene, slight inconsistencies in sgRNA representation targeting a large number of genes can masquerade as hits with negative selection survival screens. Hence, if feasible, it would be worthwhile to employ positive selection survival screen formats. One broadly applicable implementation of a positive neurodegenerative disease screen would look for factors that confer resistance to certain toxins and insults, e.g., Aβ, proteotoxic aggregates, glutamate [41], or virus attack (see below).

#### Marker selection screens

CRISPR KO, CRISPRa, and CRISPRi can also be used for marker selection screens that aim to identify gene elements affecting the expression of a specific reporter molecule. In one implementation, the reporter can be genetically engineered by replacing the coding sequence of a gene-of-interest with the coding sequence for a fluorescent or luminescent marker. This type of design may reveal upstream expression regulators. Alternatively, a cassette coding for a markers can be fused to the gene-of-interest or antibodies thereby allowing to visualize a protein-of-interest or sort cells expressing it by fluorescence-activated cell sorting (FACS) [41, 47, 70]. An innovative advance facilitating this objective offered a report which documented the specific GFP tagging of endogenous human genes using a split-GFP expression approach [71]. Rather than inserting the entire GFP coding sequence, which would require long homology arms, the authors designed a 200 nucleotide in length single-stranded DNA that can be rapidly synthesized. The latter served as the HDR template by comprising homology arms that flanked a coding sequence for a 16 amino acid GFP fragment (GFP11). When combined with the stable expression of a complementary GFP construct (GFP1–10), this strategy enabled the rapid generation of GFP-tagged human cell lines.

A common concern when using any protein tagging approach is whether the tag destabilizes and disrupts the function of its fusion partner. One also needs to consider whether the readout can indeed be measured or separated by FACS. For instance, if one would like to study a phenotype that relates to a cells’ morphology, electrophysiology, or extracellular secretome, e.g., Aβ plaques or inflammatory cytokines, the use of an arrayed screen may be preferable.

Specialized FACS techniques are available to address the frequent need of neurodegenerative disease research to work with abnormally aggregated proteins. These include pulse-shape analysis (PulSA) and ﻿flow cytometric analysis of inclusions and trafficking (FloIT) [72, 73]. These methods have been used to study GFP-fused Huntingtin exon 1 and superoxide dismutase 1 (SOD1). However, a looming concern when fusing protein domains to aggregation-prone monomers is the possibility of inadvertently influencing the pathological misfolding and aggregation process, thereby undermining the physiological relevance of the screen. Thus, a solution can be to first allow the native protein monomers to aggregate, and then use fluorescently tagged antibodies to specifically detect the aggregated structures.

Whereas antibodies can readily recognize protein aggregates on the cell surface, they are typically not able to get into the cell to bind intracellular inclusions. Hence, detecting intracellular protein aggregates with antibodies requires either the use of specifically-designed intrabodies or cell fixation and permeabilization, which may hinder genomic PCR and sequencing of the integrated sgRNAs. Thus, for these applications it may be prudent to employ an arrayed screen format, in which individual clones can be useful for immunocytochemistry because their sgRNA is already known.

Oftentimes, investigators may choose established pathological hallmarks as the reporter in a marker selection screen. In doing so, consideration need to be given to the role of such a reporter for the pathobiology underlying the disease. This can be difficult if the clinical relevance of the reporter is not well understood [74–80]. For instance, the Aβ plaque load is a poor correlate of the severity of Alzheimer’s disease. Humans can have significant amyloid burden in their brains with no or few symptoms of Alzheimer’s disease, and the progression of Aβ deposition during the course of the disease is non-linear [75]. Moreover, removing Aβ via immunization has so far failed to improve clinical outcomes [81, 82]. Similarly, levels of TDP-43 cytoplasmic inclusions in ALS patients do not correlate well with disease progression [80]. Therefore, if a marker selection screen is built on a protein aggregation event, gene products revealed by the screen may indeed contribute to the aggregation process of the respective protein. This, however, may or may not provide insights into the central pathway that underlies cell death and clinical symptoms.

#### Experimental controls

The design of controls depends largely on the screen format. Vehicle controls should be used in parallel with experimental agents if the screen uses a chemical compound to exert selection pressure. The vehicle can often be the solvent used to dissolve the selection agent, the inactive enantiomer, or some other version of the selection agent. For instance, if one wishes to study genes that render protection or sensitivity against oligomeric amyloid beta (Aβ) in the context of Alzheimer’s disease, a suitable negative control would be monomeric Aβ.

Frequently, non-targeting sgRNAs are employed as negative technical controls [47, 57, 83]. If sensitivity/resistance genes to a particular selection agent are known, one may consider sgRNAs targeting these validated genes as functional positive controls [46]. Technical controls are used at various steps during the screen. For example, one may spike in sgRNAs that have no sequence match in the host genome to assess the technical performance of amplification and NGS. Although not yet widely applied, the targeting of non-essential genes or ‘safe harbor’ regions may serve the same purpose in a negative selection survival screen [84].

#### sgRNA amplicon sequencing

Following the selection step, DNA is harvested from surviving or FACS-sorted cells, and PCR is used to amplify the sgRNA protospacer sequences within these cells using primers that pair to the constant regions in each sgRNA viral plasmid, namely the U6 promoter region upstream and the sgRNA scaffold region downstream of the protospacer.

The presence of PCR primers can sometimes create technical difficulties on some fluorescence-based sequencing platforms because the primer sequences included at the 5′ end of each amplicon will generate the exact same bases for each cycle of sequencing, lowering diversity. Hence, to improve diversity, 1–10 random bases have been added to the 5′ end of each forward primer to make them slightly different and to stagger the order of sequencing [47, 85]. In addition, reverse primers may contain unique barcodes to differentiate biological replicates and treatment groups so that samples and controls can be analyzed simultaneously, minimizing run-to-run variances. Following NGS data acquisition, normalized reads of sgRNAs are tallied in samples and controls [47, 64], and it is verified that coverage of the sgRNA library has been maintained in the controls [86].

#### Sequencing analysis, statistics and candidate gene ranking

Analysis of the NGS data obtained from a CRISPR-Cas9 functional genomics screen should reveal a skewed distribution of sgRNAs in the experimental group compared with the control, reflecting the functional selection that has occurred [87]. Several algorithms are available to identify and rank the hits. In addition to computing the fold enrichment or depletion of a sgRNA in samples versus controls, these algorithms exploit for their ranking analysis the built-in redundancy of having several sgRNAs targeting a given genomic entity [87–89].

Several of these algorithms, e.g., the Redundant siRNA Activity (RSA) and the RNAi Gene Enrichment Ranking (RIGER) algorithms, were originally developed for analyzing data from siRNA-based functional genomics screens and barcoded microarray platforms [87]. Whereas RSA evaluates the signal of all sgRNAs and assigns a *p*-value based on an iterative hypergeometric distribution formula [90], RIGER uses Kalmogorov-Smirnov statistics to calculate an enrichment score and rank the hits based on a permutation test [91]. Although there is no consensus on the best method of analysis, the more recently developed sgRNA-specific negative-binomial model-based analysis of GeCKO (MAGeCk) algorithm was reported to exhibit superior specificity and sensitivity when comparing experimental datasets [88]. A program similar to MAGeCk, named PinAPL-Py, offers a user-friendly web-based workflow [89].

#### Validation of screen results

Screens produce a ranked list of candidate genes. In order to determine which and how many of these genes contribute to the phenotype, validation is essential. Cas9-mediated off-target DNA damage may lead to false positives [57, 66]. Investigators must also consider the pleiotropic effects of genetic manipulation to genes with multiple transcription start sites [92] or multiple splice variants [50], and the observation that introns or regulatory sequences of one gene may affect the expression of another [93]. Moreover, unlike classic Mendelian diseases, neurological diseases tend to be polygenic and involve complex interactions among gene products [94, 95]. Thus, investigators need to be alert to the fact that a CRISPR-Cas9 functional genetic screen, wherein genes are knocked out, repressed, or overexpressed one at a time, cannot capture synthetic lethal outcomes.

A freely available online tool, termed CRISPulator (http://crispulator.ucsf.edu), can be used to model the impact of screen parameters on pooled screening results. The use of this algorithm can save valuable time and resources by providing a good estimation for how a large number of design choices in CRISPRi or CRISRP KO screens are predicted to affect outcomes [96].

Although a common framework has emerged for the validation of hits, specific procedures may vary depending on the experimental question. To begin with, validation is typically directed toward genomic elements that exhibited the highest ratios of enrichment or depletion, corroborated by multiple sgRNAs, thereby giving them the highest rank in the analysis. The functional significance of candidates can be further informed by whether other genes acting on the same pathway are also implicated.

The most critical validation method is to evaluate if the introduction of sgRNAs targeting gene elements-of-interest indeed recreate the selection phenotype.

With recent advances in the specificity of sgRNAs, the need to verify target engagement is usually no longer essential, so long as multiple sgRNAs directed toward the same gene element illicit the phenotype. However, if desired, genomic PCR, RT-qPCR or Western blot analyses can be undertaken to assess the functional modification to the targeted gene element [45, 47, 50].

Rescue experiments are another way to verify if a genetic entity confers the phenotype of interest. The goal is to assess if restoring the candidate’s expression in a CRISPR-Cas9 edited cell to physiological levels reverts the cell to its wild-type state [30]. For instance, if the disruption of a gene increased the expression of a reporter, restoring the gene should comparatively decrease the expression of the reporter. For a CRISPR KO or CRISPRi screen, cDNA overexpression or inducible expression may be used for rescue [97], whereas si/shRNA-mediated reduction of transcripts or CRISPR-based KO may be useful for CRISPRa screens [45]. Naturally, a challenge with this type of rescue experiments is ensuring that the restored expression level of the gene product is physiologically relevant [45].

### Notable firsts in functional CRISPR-Cas9 genetic screens

Whenever contemplating the use of a novel technology, knowledge of its previous applications can save valuable resources. This section will briefly showcase notable applications of CRISPR-Cas9-based functional genetic screens that targeted upward of 10,000 genes.

#### Genome-scale CRISPR-Cas9 functional knockout (GeCKo) screens

**Fig. 3:**
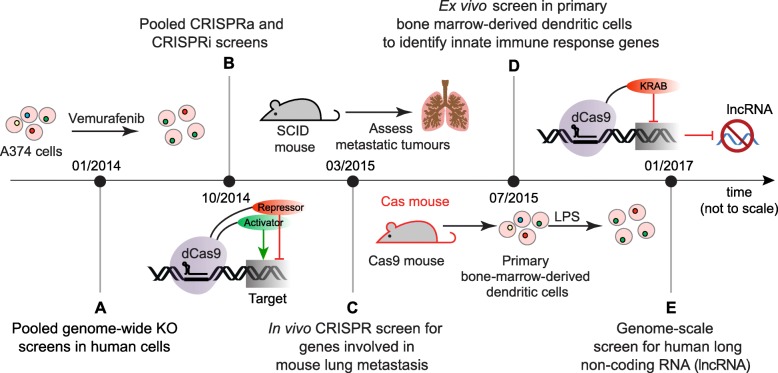
Notable firsts in the history of pooled, genome-scale CRISPR screens. **a** One of the first two CRISPR-Cas 9 KO screens searched for genes conferring vemurafenib resistance in melanoma cells [3]. **b** Subsequent CRISPR inhibition and activation (CRISPRi and CRISPRa) studies made use of deactivated Cas9 (dCas9) fused to repressor or activator domains for gene transcription modulation [50]. **c** A milestone in vivo study explored the role of a subset of genes in the evolution of metastatic tumors in an immunocompromised mouse [98]. **d** Primary cells were used in a study that employed tumor necrosis factor (Tnf) levels as a response marker to lipopolysaccharide treatment [70]. **e** A genome-scale CRISPRi screen on long, noncoding RNAs (lncRNAs) revealed that essential noncoding elements may be more cell-type specific than coding elements [99]

The first CRISPR KO screens were published back-to-back early in 2014. One of these studies was designed to identify genes that confer resistance to a potent anti-cancer drug (vemurafenib), a complication of clinical treatments that signals poor patient prognosis [3]. The other probed the genome for genes that confer resistance to 6-thioguanine, a nucleotide analog and DNA mismatch repair inhibitor that is lethal to wild-type cells [57]. Vemurafenib, an inhibitor that induces apoptosis in cells expressing mutant (V600E) serine/threonine-protein kinase B-raf (BRAF), seen in > 50% of malignant melanomas (Fig. 3). BRAF^V600E^ mutant cells were virally transduced with an sgRNA library targeting 18,080 genes before being selected with vemurafenib [3]. Post-treatment, surviving cells were assessed for sgRNA enrichment through deep sequencing. The analysis pointed toward previously validated genes NF1 and MED12, as well as novel candidates NF2, CUL3, TADA2B, and TADA1. The study provided early evidence that CRISPR KO screens can produce better consistency within top-ranking hits—indicated by lower *p* values—than RNAi screens, a conclusion that has since been supported by others [100].

The selection step at the heart of CRISPR KO screens is not limited to in vitro studies of cultured cells but can also be applied to physiologically relevant tissue environments, as showcased by a search for genes that contribute to tumor metastasis [98]. In this study, mouse non-small-cell lung cancer (NSCLC) cells were transduced in vitro with a CRISPR KO sgRNA library targeting 20,611 genes and then transplanted subcutaneously into the flanks of immunocompromised nude mice (Nu/Nu) [98]. Post-transplantation, sgRNA subsets from surviving cells in primary and metastatic tumors were compared. The sgRNA pool retrieved from primary tumors would be expected to be enriched for genes that enhance metastasis, because their functional ablation has prevented it. In contrast, the sgRNA pool from metastatic tumors would be enriched in anti-metastatic genes. The experiment provided a powerful means to interrogate the human genome for candidates influencing tumor evolution in an environment that more closely mimics the endogenous human condition.

The first CRISPR KO-based marker screen in primary cells targeted bone marrow-derived dendritic cells (BMDCs) isolated from Cas9-expressing transgenic mice. It aimed to identify genes influencing host response to pathogenic lipopolysaccharide (LPS) by assaying tumor necrosis factor (TNF), a marker of early LPS response, via intracellular staining following LPS stimulation [70]. To this end, the BMDCs were transduced with an sgRNA library targeting 21,786 genes. The study uncovered new TNF modulators and established the utility of such a screen in dissecting complex biological circuits in primary mammalian cells.

#### CRISPRa/CRISPRi

In 2014, the first genome-scale application using CRISPRa and CRISPRi, targeting 15,977 genes, was reported [50]. Earlier iterations of CRISPRi relied solely on recruiting dCas9 to sterically hinder the binding of other transcription factors [101]. This approach had produced modest transcriptional suppression, but it was insufficient for genome-scale studies. To overcome this limitation, the Kruppel-associated box (KRAB) repression effector domain was fused to dCas9 [21, 50]. This study used a chimeric cholera/diphtheria fusion toxin (CTx-DTA) model and established the robustness of the method.

Early implementations of CRISPRa were similar to CRISPRi in that they relied on fusing a single transcriptional activation domain, e.g., the herpes virus derived VP64 domain, to dCas9 [102]. More recent optimizations have shown that activation efficacy can be further improved by engineering a synergistic activation mediator complex (SAM) including additional activation domains to the original dCas9-VP64 fusion. A successful implementation of this SAM-based approach sought to identify among > 20,000 genes those that confer resistance to a BRAF inhibitor [23].

Alternative CRISPRa derivatives have been developed that also produce robust transcriptional activation, e.g., one that uses a protein scaffold system made up of repeating peptide arrays fused to a single-chain variable fragment (ScFv) antibody [23, 103, 104].

#### Probing the non-coding genome

Most functional genomic studies to date have focused on the small subset of the genome that encodes proteins. More recently, interest has shifted toward interrogating the noncoding genome, a largely unexplored domain increasingly understood to be critical to health and disease [105]. Following on the heels of a more focused screen that tiled sgRNA across > 700 kb of noncoding region surrounding three specific genes [106], one of the first pooled, genome-scale CRISPRi screens that targeted long noncoding RNAs (lncRNA) aimed to uncover novel genomic elements essential for cell growth. To this end, it targeted 16,401 lncRNAs exceeding 200 bp in length in 7 transformed and non-transformed human cells [99]. The screen monitored cell growth across the different lines and revealed 499 lnRNAs whose presence was essential for robust cell growth. Interestingly, essential lncRNA hits differed among the cell lines tested, highlighting the subtleties of cell-type-specific complexities in the human noncoding genome.

### CRISPR-Cas9-based functional genetic neurological disease screens to date

To date, few CRISPR-Cas9 based screens have been reported in the neurological disease field, presumably in part because human neurological diseases are primarily studied in non-dividing brain cells. This section showcases five CRISPR-Cas9 functional screens that interrogated the biology of neurodegenerative disease proteins or shed light on host factors that interact with Zika viruses (Table 1).

**Table 1:** Milestone neurological disease studies that made use of genome-scale CRISPR-Cas9 screens

Disease	Screen objective	Screen type	Methodology	Results	Reference
ALS	To find regulators of SQSTM1/p62	Activation	Lentiviral transduction of sgRNA library into human neuroglioma H4 cells expressing GFP-tagged SQSTM1 and Cas9. Selection through FACS. A mini-pool screen followed to verify top hits.	Identified the MTOR signaling pathway and the entire macroautophagy machinery as key regulators of SQSTM1. Also uncovered HNRNPM, SLC39A14, SRRD, PGK1, and the ufmylation cascade as modulators.	[107]
PD	To find transcriptional networks that protect against alpha-synuclein toxicity	Activation	Doxycycline (Dox)-inducible (Tet-ON) dCas9-VP64 expression cassette was integrated into yeast cells expressing YFP-tagged alpha-synuclein. Cells were transformed with sgRNA library and selected for survival. Validation in yeast and SHSY5Y cells.	Identified crisprTFs that were protective against alpha-synuclein toxicity that modulate protein quality control, ER/Golgi trafficking, lipid metabolism, mitochondrial function, and stress response.	[30]
PD	To elucidate the effects of cellular PARKIN abundance on downstream processes	Knockout	Lentiviral transduction of sgRNA library into human HEK-derived JumpIN TI 293 cells that express endogenous GFP-tagged PARKIN. Selection through FACS. Top hits were verified in iPSC iNGN cells.	Identified genes that regulate PARKIN gene expression positively and negatively. Specifically, transcriptional repressor THAP11 can repress PARKIN and impact pUb accumulation.	[28]
ALS	To find genetic modifiers of C9orf72 peptide repeat toxicity	Knockout	Lentiviral transduction of sgRNA library into human myelogenous leukemia K562 cells. Treatment with synthetic or lentivirally transduced DPR proteins in two separate screens. Validation in subset screen based on mouse primary neurons. Top hits from both screens were validated in mouse dorsal root ganglion axons and iPSCs from patients.	Uncovered potent candidate modifiers of DPR toxicity. Specifically, TMX2 was observed to modify DPR toxicity and exhibited promise as a therapeutic target.	[31]
Zika virus	To find host encoded proteins that mediate Zika virus infection	Knockout	Lentiviral transduction of sgRNA library into human iPSC derived neuroprogenitor cells. Zika virus infection causes majority of cells to die 48 h postinfection. Validation in a subset screen based on human iPSC and ESC cells validated top-ranked hits from initial screen.	Identified gene products with roles in heparan sulfation, ER translocation and proteostasis, Golgi-based glycosylation and the cellular response to interferon, as mediators of Zika virus-dependent cell death	[108]

SQSTM1 is a gene involved in autophagy and suspected to play a role in neurodegenerative diseases, including amyotrophic lateral sclerosis (ALS). More specifically, SQSTM1 regulates protein degradation pathways and has been found associated with protein aggregates [109–111]. In 2016, a positive marker selection screen was undertaken to identify proteins that control steady-state expression levels of SQSTM1 (also known as p62) [107]. Using lentiviral expression, a pooled sgRNA library was transduced into human neuroglioma cells (H4) expressing a GFP-tagged SQSTM1 reporter and Cas9 [107]. Cells were FACS sorted based on their GFP-SQSTM1 expression levels, and their genome-embedded sgRNAs were sequenced to generate a ranked list of candidate SQSTM1 regulators. To validate hits from the screen, researchers followed up with a small-scale pooled screen that targeted the top 300 candidates in the same neuroglioma cell model. These analyses shortlisted the mammalian target of rapamycin (MTOR) complex 1, the macroautophagy machinery, the ubiquitin fold modifier 1 and functionally interlinked proteins as contributing to SQSTM1 steady-state expression levels.

Another neurodegenerative disease-themed screen, the first in a yeast model [30], sought to uncover transcriptional networks that protect against toxicity elicited by alpha-synuclein aggregation in Lewy bodies, a central pathological hallmark of Parkinson’s disease (PD). The study used a derivative technique—Perturbing Regulatory Interactions by Synthetic Modulators (PRISM)—to study genetic interactions in a transcriptional network. PRISM uses randomized sgRNAs (i.e., an oligo library encoding 20-mer randomized nucleotides) and CRISPR-dCas9 transcription factors (crisprTFs) to perturb the transcriptome and find pathways or gene networks that promote cell survival. To generate the model, a dCas9-VP64 expression cassette was integrated into yeast cells expressing YFP-tagged alpha-synuclein. Cells were then transformed with the randomized sgRNA library, positively selected for survival, and sequenced for top hits. The screen identified several sgRNAs of interest. One of them rescued the screen yeast strain from alpha-synuclein toxicity but—perhaps surprisingly—had no specific sequence match in the yeast genome. It therefore was most likely acting through off-target binding to one or more genes. The authors showed that the presence of this sgRNA caused transcriptional perturbations exceeding two-fold changes to 114 genes involved in regulating protein quality control, ER/Golgi trafficking, lipid metabolism, mitochondrial function, and stress responses. The results were subsequently cross-validated in differentiated human neuroblastoma cells (SH-SY5Y).

Another study sought to elucidate genes that influence the cellular abundance of PARKIN, a gene implicated in PD known to affect downstream mitophagy pathways [28]. It employed a positive marker selection screen design in HEK-derived JUMPIN TI 293 cells that expressed a GFP-PARKIN fusion from the endogenous PARKIN locus. The screen identified 53 positive or negative regulators of GFP-PARKIN, including a transcription factor, THAP11, that was subsequently validated to repress PARKIN expression. The authors verified their results in human neuroblastoma SH-SY5Y cells and in induced pluripotent stem cells (iPSCs) that were differentiated into excitatory neurons [112].

The use of CRISPR-Cas9 screens in the neurodegenerative disease field was further refined by a group that sought to find genetic modifiers of C9orf72 dipeptide repeat toxicity using a CRISPR KO screen [31]. Mutations in the C9orf72 gene are the most common genetic cause of ALS; dipeptide repeat (DPR) proteins produced by these mutations accumulate in patients’ neurons and are suspected to be the cause of neuronal toxicity in ALS. The CRISPR KO screen was conducted in Cas9-expressing human myelogenous leukemia cells (K562) using lentiviral expression of the sgRNA library, and synthetic DPR proteins were introduced exogenously to the cells to model accumulation of DPR proteins in ALS. Deep sequencing was then used to identify sgRNAs that were protective, sensitizing, or neutral towards DPR toxicity. In order to evaluate the top hits in a more disease-relevant context, the group also undertook a secondary CRISPR KO screen in primary mouse cortical neurons that uncovered potent modifiers of DPR toxicity, e.g., TMX2. Lowering TMX2 levels produced a strong protective effect in mouse dorsal root ganglion axons and iPSCs from C9orf72-ALS patients. To our knowledge, this study was the first to conduct a CRISPR-Cas9 screen in primary neurons. Currently, CRISPR-Cas9 screens using iPSC-derived human neurons from controls and patients are being developed that hopefully will provide meaningful insights into the pathobiology of neurodegeneration [41]. In fact, a first leading-edge manuscript describing the use of this paradigm for a series of CRISPRi-based functional genetic screens was published most recently. The study revealed in three separate screens genes essential for neuronal survival, single-cell transcriptomic states or morphology [113].

A line of neurological disease investigation that has put CRISPR-Cas9 functional genetic screens to particularly rewarding use has focused on the interactions of Zika viruses (and a small number of other viruses) with human cells. Because this body of work, comprising a half dozen of papers published since 2016 [108, 114–116], has recently been extensively reviewed [117–119], we will focus here on what is, to our knowledge, the first positive selection survival screen that utilized human neural cells to study Zika-host cell factors [108]. Human neuroprogenitor precursors are particularly susceptible to Zika virus infection, supporting the decision of the authors to base their study on neuroprogenitor cells obtained through differentiation of wild-type human iPSCs. The study made use of a lentiviral library of 187,535 sgRNAs, which targeted 18,663 protein-coding human genes and 1503 intergenic targeted and nontargeting control sgRNAs. As expected, Zika virus infection led to cell death in most cells. The small population of surviving cells harbored sgRNAs that targeted genes encoding proteins with roles in heparan sulfation, ER translocation and proteostasis, Golgi-based glycosylation and the cellular response to interferon. A more focused validation screen, undertaken with human neuroprogenitors cells from two different genetic backgrounds, iPS-wt5 and WIBR3 ESCs, validated the top-ranked hits from the initial genome-scale screen.

## Conclusions

**Fig. 4:**
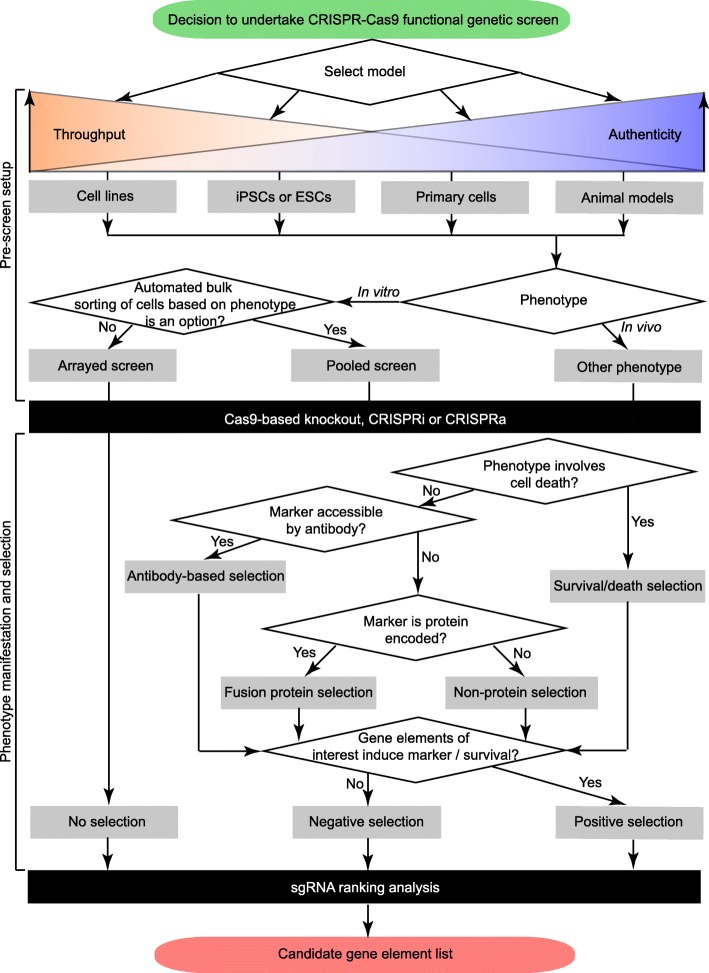
Key considerations in choosing a screening method. Each research question poses a new set of challenges that need to be considered when selecting an appropriate screening method. This flowchart is intended to provide some initial guidance for investigators embarking on a CRISPR-Cas9 functional genomics screen regarding the choice of model and the type of screens that may be employed to address the neurological disease research question at hand

Genome-scale CRISPR-Cas9 functional analyses offer a powerful novel modality for interrogating genomic elements. Since its introduction in 2014, a series of milestone reports established that this technology can deliver unprecedented signal-to-noise and high-quality functional hits. When combined with other orthogonal methods of interrogating protein function at a systems scale (e.g., mass spectrometry), this technology can add valuable functional insights that may take years to establish using conventional approaches. It is reasonable to expect that the methodology and toolbox available to undertake these screens will continue to evolve in tandem with improved systems for viral delivery and CRISPR-Cas9 genome editing [120]. This review was written with the intent to provide some initial guidance for neurological disease investigators embarking on a CRISPR-Cas9 functional genomics screen (Fig. 4). We hope it will entice researchers to adopt this powerful technology to address some of the most pressing unanswered questions related to the pathobiology and mechanisms of cell death underlying this group of diseases.

## Data Availability

Not applicable.

## Abbreviations

AAV: Adeno-associated virus
CRISPR: Clustered regularly interspaced short palindromic repeats
CRISPRa: CRISPR activation
CRISPRi: CRISPR inhibition
DSB: Double-stranded break
EGFP: Enhanced green fluorescent protein
ESCs: Embryonic stem cells
FACS: Fluorescence-activated cell sorting
HDR: Homology-directed repair
iPSCs: Induced pluripotent stem cell
KO: Knockout
MOI: Multiplicity of infection
NGS: Next-generation sequencing
NHEJ: Non-homologous end joining
PAM: Protospacer adjacent motif
SAM: Synergistic activation mediator
TALENs: Transcription activator-like effector nucleases
ZFN: Zinc finger nucleases
